# Identification of BANF1 as a novel prognostic biomarker in gastric cancer and validation via *in-vitro* and *in-vivo* experiments

**DOI:** 10.18632/aging.205461

**Published:** 2024-01-22

**Authors:** Yuanmin Xu, Xu Wang, Weiwei Yuan, Ling Zhang, Wei Chen, Kongwang Hu

**Affiliations:** 1 Department of General Surgery, First Affiliated Hospital of Anhui Medical University, Hefei 230022, China; 2 Department of General Surgery, Anhui Public Health Clinical Center, Hefei 230022, China; 3 Department of Immunology, School of Basic Medical Sciences, Anhui Medical University, Hefei 230022, China; 4 Department of General Surgery, Fuyang Affiliated Hospital of Anhui Medical University, Fuyang 236000, China

**Keywords:** gastric cancer, barrier to autointegration factor 1, overall survival, TCGA, GEO

## Abstract

Gastric cancer (GC) is a widespread malignancy characterized by a notably high incidence rate and an unfavorable prognosis. We conducted a meticulous analysis of GC high-throughput sequencing data downloaded from the Gene Expression Omnibus (GEO) repository to pinpoint distinctive genes associated with GC. Our investigation successfully identified three signature genes implicated in GC, with a specific focus on the barrier to autointegration factor 1 (BANF1), which exhibits elevated expression across various cancer types, including GC. Bioinformatic analysis has highlighted BANF1 as a prognostic indicator for patients with GC, with direct implications for immune cell infiltration. To gain a more comprehensive understanding of the significance of BANF1 in GC, we performed a series of *in vitro* experiments to confirm its high expression in GC tissues and cellular components. Intriguingly, the induction of BANF1 knockdown resulted in a marked attenuation of proliferation, migratory capacity, and invasive potential in GC cells. Moreover, our *in vivo* experiments using nude mouse models revealed a notable impediment in tumor growth following BANF1 knockdown. These insights underscore the feasibility of BANF1 as a novel therapeutic target for GC.

## INTRODUCTION

GC poses a significant global health challenge within the gastrointestinal tract. In 2020 alone, the global incidence of GC surpassed 1,089,103 cases, leading to the unfortunate demise of over 760,000 individuals [1]. *Helicobacter pylori* infection stands out as a major contributing factor to GC [2]. Diagnostic strategies, such as computed tomography, endoscopy, and histopathological examination, form the primary tools for GC detection. Surgical intervention remains pivotal for early-stage GC, while unresectable cases necessitate a multimodal approach combining symptomatic management, chemotherapy, and targeted therapies to enhance both quality of life and survival periods [3, 4]. The intrinsic heterogeneity of GC, as classified by The Cancer Genome Atlas (TCGA) into four subtypes, poses a formidable challenge, emphasizing the need for personalized treatment strategies [5]. Molecular biology investigations utilizing high-throughput sequencing hold promise in uncovering novel therapeutic targets for a more effective GC treatment landscape.

Barrier to autointegration factor 1 (BANF1), predominantly situated in the inner nuclear membrane, plays a crucial role in fundamental biological processes [6]. In the context of DNA repair, BANF1 emerges as a central figure in resolving DNA double-stranded breaks, safeguarding cells from demise caused by the exposure of double-stranded DNA to the cytoplasm during nuclear rupture [7]. Its influence on the cell cycle, intricately linked to cell mitosis initiation and cessation in phosphorylated and dephosphorylated states, further underscores its significance [8]. The relevance of BANF1 to human health is exemplified in premature aging syndromes, as indicated by previous studies [9]. Recent research has linked elevated BANF1 expression to breast cancer, correlating with lymph node positive and pathological staging, and to differentiation in esophageal tumor and influencing the proliferation, migration, and invasion of cervical cancer [10–12]. These findings suggest the potential of BANF1 as a biomarker and therapeutic target in various malignant neoplasms, including GC.

Our study aimed to comprehensively analyze transcriptome data from GC samples obtained from GEO and TCGA repositories. We sought to elucidate the pivotal role of BANF1 in GC pathogenesis through bioinformatic analyses, assessing its expression pattern, diagnostic utility, prognostic implications, and relevance within the immune milieu. *In vitro* experiments were conducted to validate BANF1 expression in gastric cells and understand its impact on their proliferative, migratory, and invasive properties. The investigation in nude mice aimed to assess the effect of BANF1 on tumorigenesis. This study was driven by the dual objectives of identifying prospective GC biomarkers and empirically validating BANF1 as a therapeutic target for this malignancy.

## RESULTS

### Differentially expressed genes (DEGs) in GC

**Figure 1:**
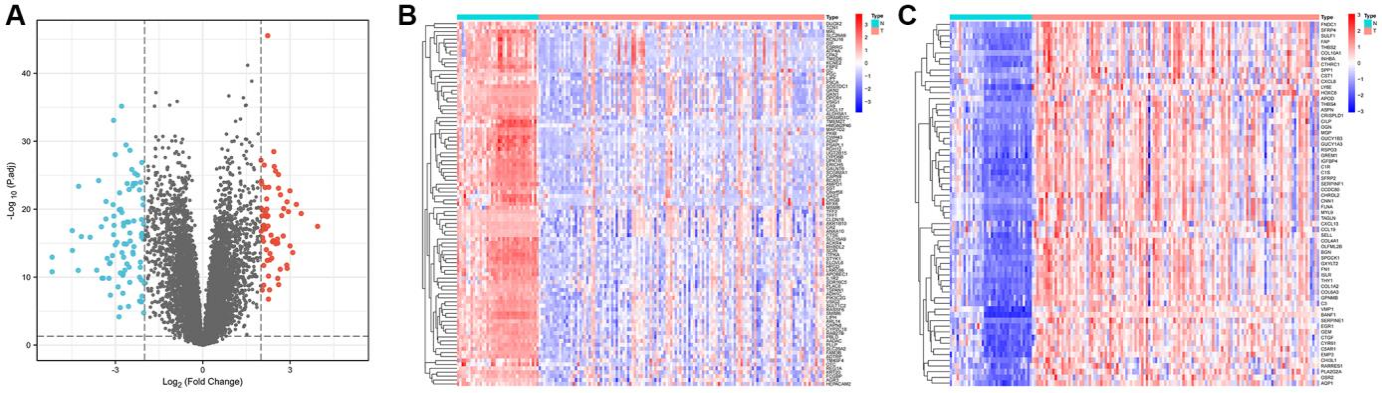
**Identification of differentially expressed genes in gastric cancer and paracancerous tissues.** (**A**) Volcano plot of 157 differentially expressed genes (|Log2FC| > 2, *p* < 0.01). (**B**) Heatmap of 93 down-regulated genes expressed in gastric cancer tissues. (**C**) Heatmap of 64 expressed up-regulated genes in gastric cancer tissues.

We obtained gene expression data for a cohort of 162 GC samples from the GSE54129 and GSE118916 datasets. The principal component analysis (PCA) plots depict gene expression in the GSE54129 and GSE118916 datasets before and after removal of batch effect. Supplementary Figure 1A illustrates the PCA results before batch-effect removal, where the two datasets were initially separated. Subsequently, the PCA plot after batch-effect removal demonstrated an intersection of gene expression data from the two datasets (Supplementary Figure 1B), enabling further analysis. By examining the expression patterns across tumor and normal samples, we successfully identified 157 DEGs characterized by |Log2FC| >2 and *p* < 0.01, as shown in Figure 1A. Among these, 93 genes were downregulated, while 64 were upregulated, visually depicted in Figure 1B, 1C.

### Identification of hub genes through weighted correlation network analysis (WGCNA)

**Figure 2:**
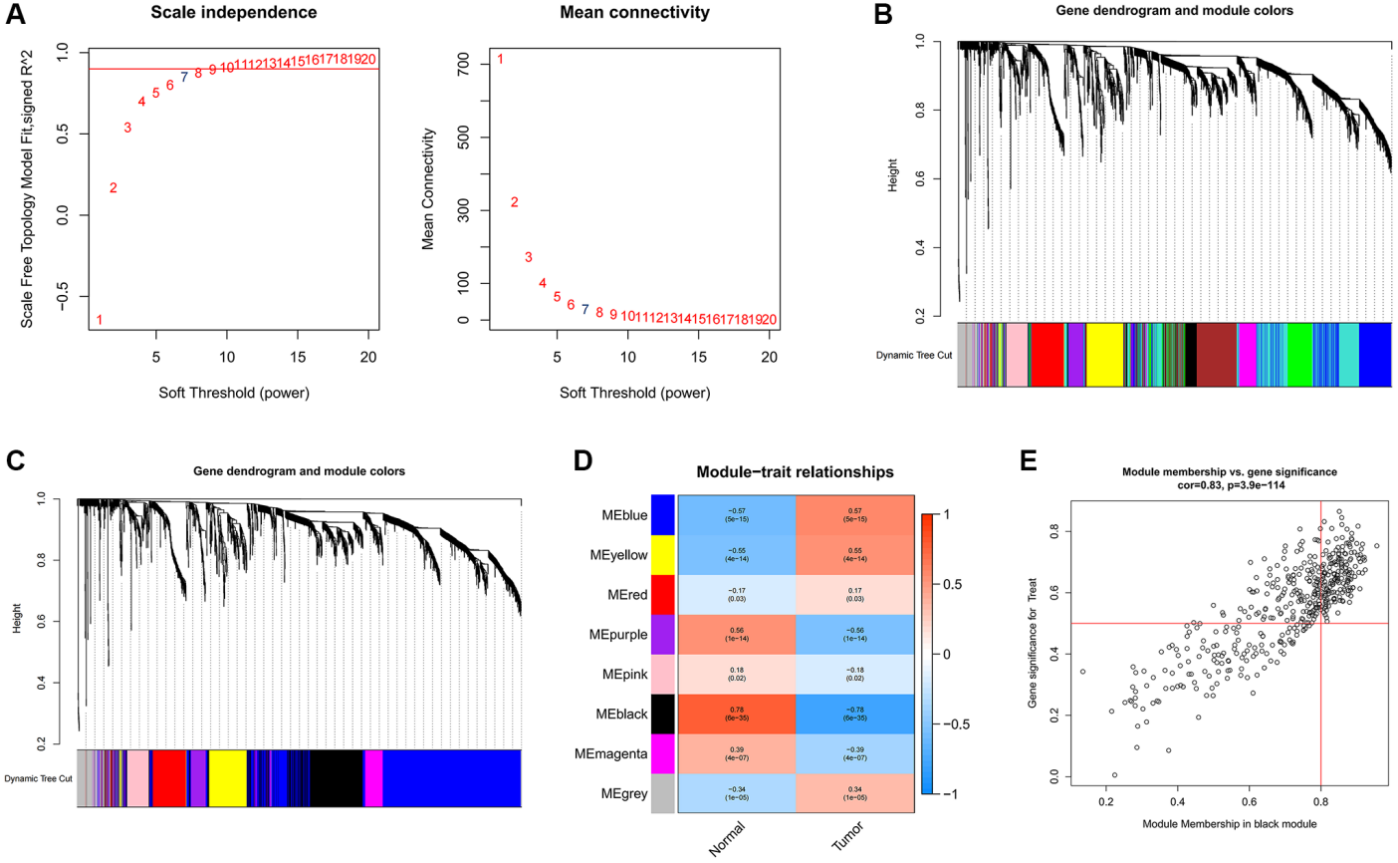
**Identification of hub genes in gastric cancer using the WGCNA algorithm.** (**A**) The soft threshold is determined by function. The left panel shows the relationship between the scale-free network evaluation metric R^2^ and the soft threshold, and the right panel shows the relationship between average connectivity and the soft threshold. (**B**) Dendrogram of gene clustering and different colored modules. (**C**) Gene clustering dendrogram obtained by merging similar modules. (**D**) Correlations between merged modules and clinical traits, correlation coefficients and p-values are shown in the corresponding modules of different colors. (**E**) Scatterplot of MM and GS in the black module.

To uncover pivotal genes of significance within GC samples, we conducted a comprehensive WGCNA. The optimal soft threshold was selected as 7, as depicted in Figure 2A, led to the establishment of gene clustering results marked by a threshold ensuring at least 60 genes per module, resulting in 11 distinct modules (Figure 2B). These modules underwent consolidation with a clipping height of 0.25, ultimately amalgamating into eight unique modules (Figure 2C). Subsequently, we computed the correlation coefficients between each gene module and samples from both normal and GC contexts. Upon rigorous assessment of correlation coefficients and associated *p*-values, we observed that the black module exhibited a noteworthy correlation with clinical traits, characterized by a correlation coefficient of 0.78 and *p* < 0.001 (Figure 2D). Figure 2E contains 444 data points, visually represents the relationship between gene significance (GS) and module membership (MM). The horizontal axis signifies MM, while the vertical axis represents GS. Applying stringent criteria (MM >0.8 and GS >0.5), we identified a cadre of 213 hub genes.

### Machine learning screens for disease characterizing genes

**Figure 3:**
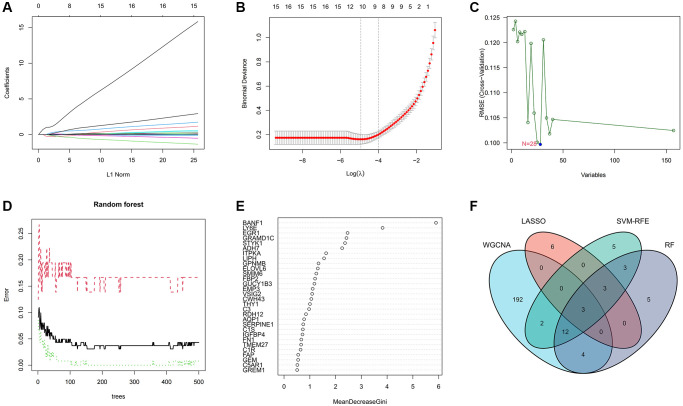
**Machine learning screens for disease characterizing genes.** (**A**, **B**) LASSO regression analysis screening variables. (**C**) Cross-validation error rate graph based on SVM-RFE. (**D**, **E**) Genes were scored using a random forest algorithm to rank genes by importance algorithm. (**F**) VEEN graph to obtain the intersection of key genes screened by the 4 methods.

To delineate the key genes linked to GC, we conducted a rigorous analysis employing least absolute shrinkage and selection operator (LASSO), support vector machine-recursive feature elimination (SVM-RFE), and random forest (RF) methodologies. Within the LASSO algorithm framework, we carefully selected optimal lambda parameters and executed a 10-fold cross-validation procedure, ultimately pinpointing a cohort of 12 genes, as illustrated in Figure 3A, 3B. The SVM-RFE algorithm identified the optimal subset of genes by pinpointing points corresponding to the lowest error rate, resulting in the selection of 28 genes, as demonstrated in Figure 3C. Furthermore, the RF algorithm provided a comprehensive assessment of each gene, ranking them based on their relative importance levels, as depicted in Figure 3D, 3E. Converging the outcomes derived from WGCNA, LASSO, SVM-RFE, and RF algorithms, we conducted an intersection analysis using a Venn diagram. This meticulous process led to the identification of three distinct genes – BANF1, ADH7, and TMEM27 – collectively characterized as hallmark genes associated with GC (Figure 3F).

### Diagnostic evaluation of three characterizing genes

**Figure 4:**
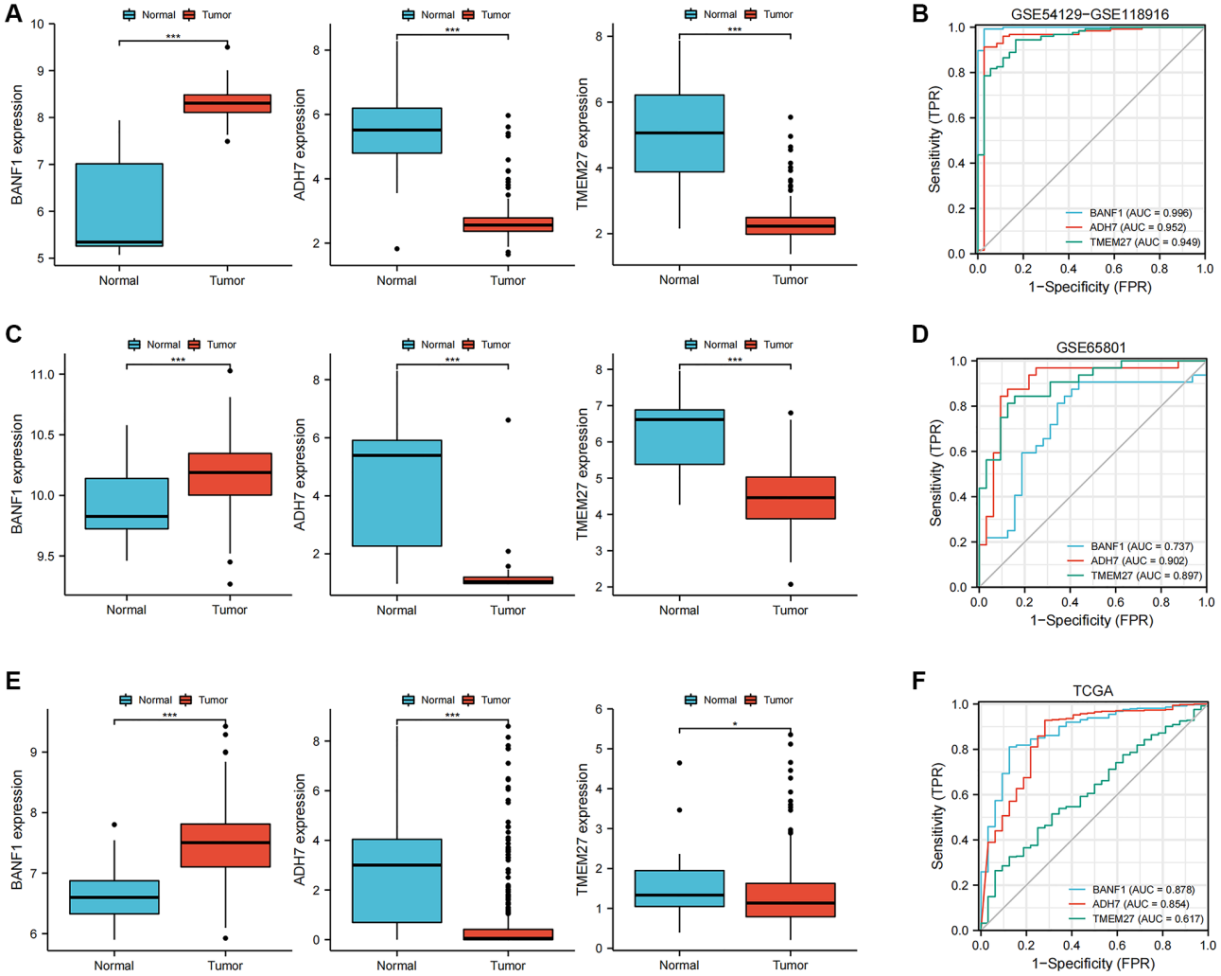
**Expression and diagnostic value of characterized genes.** (**A**) The box plots showed the expression of three GC characteristic genes (BANF1, ADH7, TMEM27) in GSE54129-GSE118916 dataset. (**B**) ROC curves were used to evaluate the diagnostic efficacy of three GC characteristic genes (BANF1, ADH7, TMEM27) in GSE54129-GSE118916 dataset. (**C**) The box plots showed the expression of three GC characteristic genes (BANF1, ADH7, TMEM27) in GSE65801 dataset. (**D**) ROC curves were used to evaluate the diagnostic efficacy of three GC characteristic genes (BANF1, ADH7, TMEM27) in GSE65801 dataset. (**E**) The box plots showed the expression of three GC characteristic genes (BANF1, ADH7, TMEM27) in TCGA dataset. (**F**) ROC curves were used to evaluate the diagnostic efficacy of three GC characteristic genes (BANF1, ADH7, TMEM27) in TCGA dataset. ^*^p < 0.05, ^***^p < 0.001.

The expression levels and diagnostic efficacy of three key genes, namely BANF1, ADH7, and TMEM27, were thoroughly examined across diverse datasets. This assessment utilized the Wilcoxon test and receiver operating characteristic (ROC) analysis. Notably, BANF1 exhibited significant upregulation in the GSE54129-GSE118916 dataset, GSE65801, and TCGA datasets, while ADH7 and TMEM27 showed downregulation (Figure 4A, 4C, 4E; *p* < 0.05). In the GSE54129-GSE118916 dataset, the area under the curve (AUC) values for BANF1, ADH7, and TMEM27 were 0.996, 0.952, and 0.949 respectively (Figure 4B). For the GSE65801 dataset, the AUC values for BANF1, ADH7, and TMEM27 were 0.737, 0.902, and 0.897 respectively (Figure 4D). In the TCGA dataset, AUC values of 0.878, 0.854, and 0.617 were assigned to BANF1, ADH7, and TMEM27, respectively (Figure 4F).

### Prognostic evaluation of three characterizing genes

**Figure 5:**
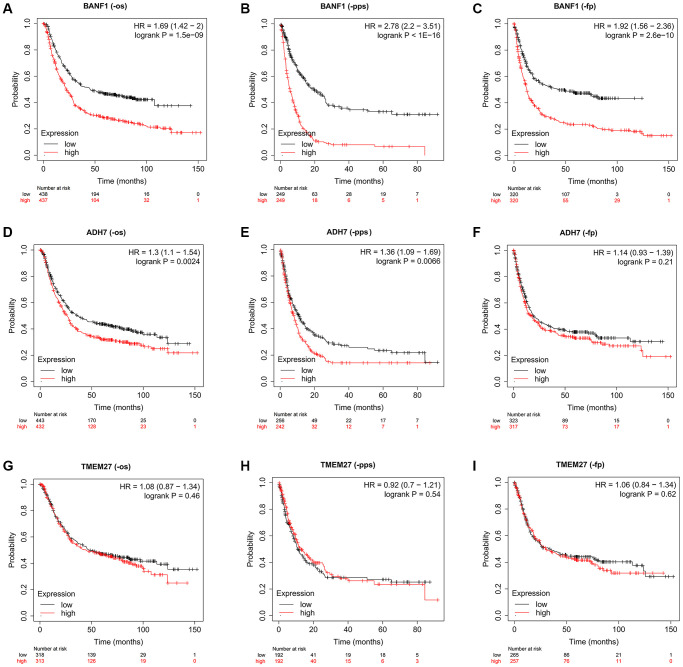
**The prognostic value of three characteristic genes.** (**A**–**C**) OS, PPS, FP km survival curves between high and low expression groups of BANF1. (**D**–**F**) OS, PPS, FP km survival curves between high and low expression groups of ADH7. (**G**–**I**) OS, PPS, FP km survival curves between high and low expression groups of TMEM27.

To assess the prognostic implications of BANF1, ADH7, and TMEM27, we utilized the Kaplan–Meier plotter database to construct survival curves. Compared with patients with low BANF1 expression, GC patients with high BANF1 expression level had significantly lower overall survival (OS), progression-free survival (FP), and post-progression survival (PPS) than GC patients with low BANF1 expression. (Figure 5A–5C, *p* < 0.001). Patients in the ADH7 high-expression cohort were distinctly associated with significantly lower OS and PPS compared to those in the low-expression group (Figure 5D, 5E, *p* < 0.01). However, there was no significant difference between the expression of ADH7 and FP in GC patients (Figure 5F, *p* > 0.05). Intriguingly, TMEM27 expression levels showed no substantive disparities in OS, FP, or PPS between the high- and low-expression groups (Figure 5G–5I, *p* > 0.05).

### Expression of BANF1 in different cells and its relationship with tumor immunity

**Figure 6:**
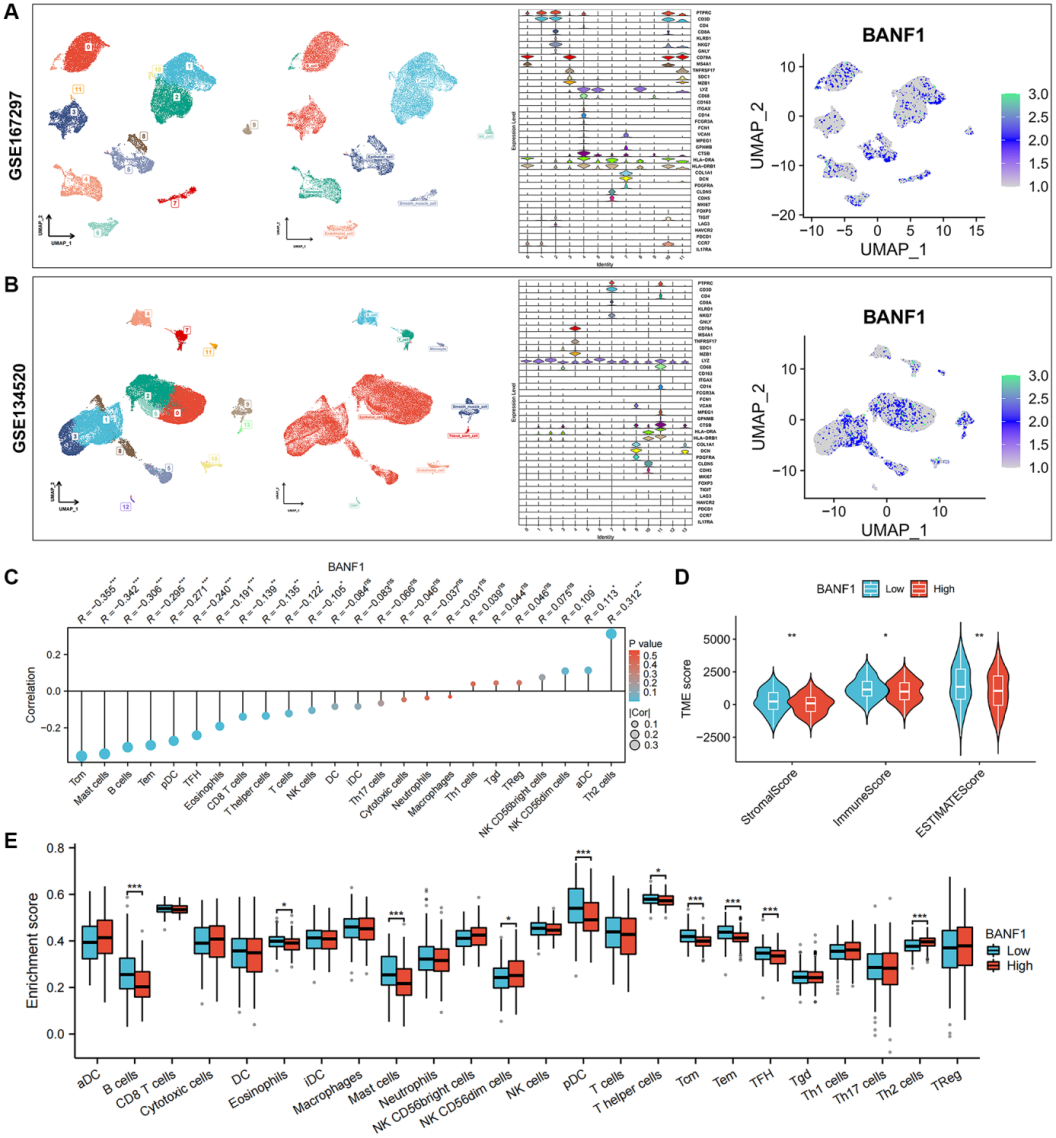
**Single-cell expression of BANF1 in gastric cancer and its immune correlation.** (**A**, **B**) Validation of BANF1 expression in different cell types in two gastric cancer single cell datasets (GSE167297, GSE134520). From left to right, cell clustering plot, cell annotation plot, violin plot of cellular signature genes expressed in different cell clusters, and BANF1 expression plot in different cells. (**C**) Lollipop plot of BANF1 expression correlating with immune cells. (**D**) Stromal score, immune score, ESTIMATE score violin plots between high and low BANF1 expression groups. (**E**) Box line plot showing immune infiltration between high and low BANF1 expression groups. ns means no statistical difference, ^*^p < 0.05, ^**^p < 0.01, ^***^p < 0.001.

We obtained single-cell RNA sequencing (scRNA-seq) datasets, specifically GSE167297 and GSE134520, from Tumor Immune Single Cell Center (TISCH) database. Using the t-distributed stochastic neighbor embedding (t-SNE) algorithm, we conducted a comprehensive clustering analysis, resulting in 12 distinct cell clusters within the GSE167297 dataset. In the GSE134520 dataset, a total of 14 cell clusters were delineated. Subsequent cell annotations were anchored in the expression patterns of diverse cellular feature genes. Notably, the uniform manifold approximation and projection (UMAP) plot revealed the ubiquity of BANF1 expression across all cell types, as shown in Figure 6A, 6B. Gene set enrichment analysis (GSEA) showed that BANF1 expression was associated with the cell cycle, Huntington's disease, olfactory transduction, spliceosomes, and systemic lupus erythematosus (Supplementary Figure 2). Subsequent correlation analysis between BANF1 expression data and immune infiltration data revealed a noteworthy negative correlation. Specifically, BANF1 expression exhibited an inverse relationship with the presence of various immune cell types, including Tcm, Mast cells, B cells, Tem, pDC, TFH, Eosinophils, CD8+ T cells, T helper cells, T cells, and NK cells, within GC tissues (Figure 6C; *p* < 0.05). In the realm of tumor microenvironment (TME) analysis, we ascertained that the immune score, stromal score, and Estimation of Stromal and Immune cells in Malignant Tumor tissues using Expression data (ESTIMATE) score in the BANF1 high-expression cohort markedly lagged those in the BANF1 low-expression group, as exemplified in Figure 6D (*p* < 0.05). Moreover, the investigation of disparities in immune infiltration between the high and low BANF1 expression groups underscored a reduction in the abundance of various immune cell populations, including B cells, Eosinophils, Mast cells, pDC, T helper cells, Tcm, Tem, and TFH, within the high BANF1 expression group, as elucidated in Figure 6E (*p* < 0.05). In addition, we investigated the effect of BANF1 on immunotherapy. The results showed that BANF1 was significantly associated with immunotherapy efficacy in the IMvigor210 cohort (Supplementary Figure 3, *p* < 0.01).

### BANF1 expression is elevated in GC cell lines and tissues

**Figure 7:**
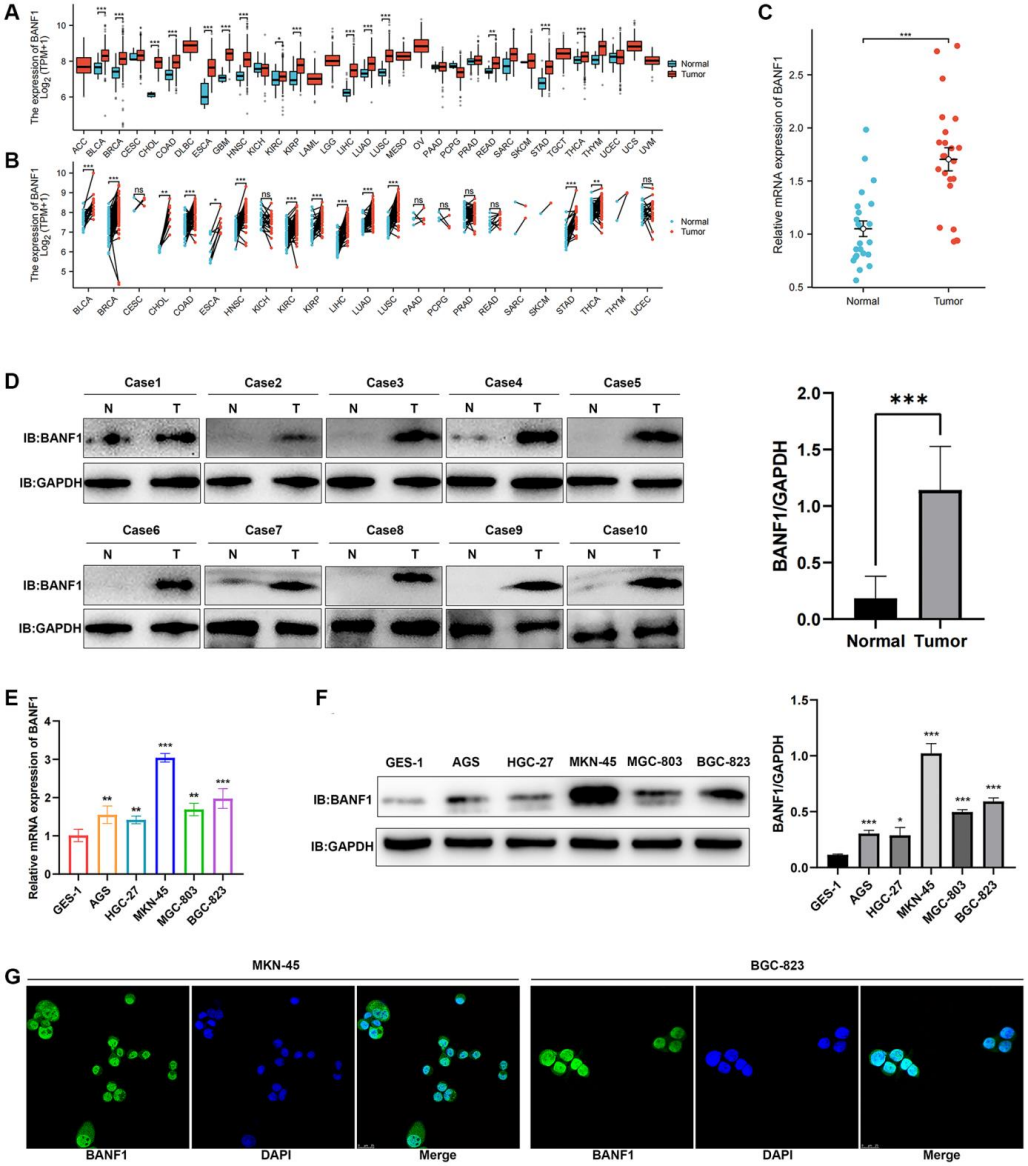
**Expression and localization of BANF1.** (**A**) BANF1 expression among pan-cancer unpaired samples in the TCGA database. (**B**) BANF1 expression among paired samples of pan-cancer in the TCGA database. (**C**) RT-qPCR detection of BANF1 mRNA expression in 23 pairs of GC tissues and paired paracancerous tissues. (**D**) WB detection of BANF1 protein expression in 10 pairs of GC tissues, and the difference of gray values between gastric cancer tissues (*n* = 10) and adjacent tissues (*n* = 10) were compared. (**E**) RT-qPCR was used to detect the expression of BANF1 mRNA in GC cell lines (AGS, HGC-27, MKN-45, MGC-803, BGC-823) and gastric mucosal epithelial cell line (GES-1), respectively. (**F**) The expression of BANF1 protein in 6 cell lines was detected by WB, and the gray values of gastric cancer cell lines (AGS, HGC-27, MKN-45, MGC-803, and BGC-823) and gastric epithelial cells (GES-1) were compared. (**G**) Immunofluorescence staining showed the expression and localization of BANF1 protein in MKN-45 and BGC-823 cells. All experiments were repeated at least three times. ns means no statistical difference, ^*^p < 0.05, ^**^p < 0.01, ^***^p < 0.001.

We conducted a thorough analysis of BANF1 expression across all cancer types within the TCGA database. The results revealed a significant upregulation of BANF1 expression in various cancers, totaling 15, including GC, when analyzing unpaired samples, as depicted in Figure 7A. A parallel analysis of paired samples showed a similar pattern, with BANF1 expression significantly increased in 13 cancers, once again including GC, as shown in Figure 7B. Examining mRNA and protein expression levels, our investigation revealed a marked increase in BANF1 within cancer tissues compared to para-cancerous tissues, as demonstrated in Figure 7C (*n* = 23; *p* < 0.001) and Figure 7D (*n* = 10; *p* < 0.001). Furthermore, both the mRNA and protein expression levels of BANF1 were conspicuously elevated in various GC cell lines, namely AGS, HGC-27, MKN-45, MGC-803, and BGC-823, in comparison to a Gastric mucosal epithelial cell line (GES-1), as illustrated in Figure 7E, 7F (*p* < 0.05). The expression level of BANF1 was the highest in MKN-45 and BGC-823 cell lines. Immunofluorescence staining on MKN-45 and BGC-823 cell lines delineated the predominant localization of BANF1 protein within the nucleus, with limited presence in the cytoplasm, as illustrated in Figure 7G.

### Knockdown of BANF1 inhibits proliferation, migration, and invasion of GC cells

**Figure 8:**
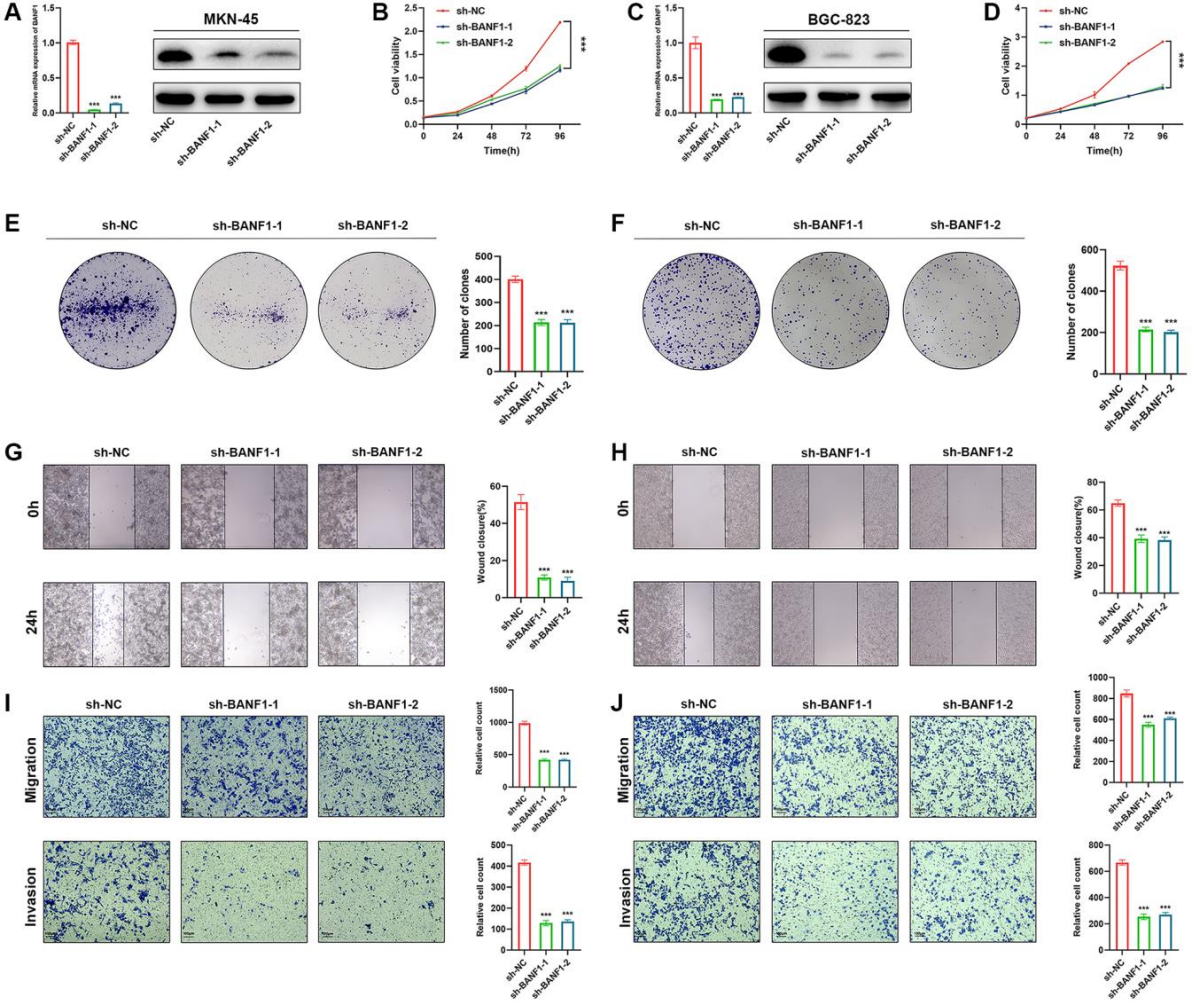
**Knockdown of BANF1 inhibits the proliferation, migration and invasion ability of GC cells in vitro.** (**A**) Knockdown efficiency of MKN-45 cell line was detected using RT-qPCR and WB. (**B**) CCK8 assay was used to detect the effect of BANF1 knockdown on the viability of MKN-45 cell line. (**C**) Knockdown efficiency of BGC-823 cell line was detected using RT-qPCR and WB. (**D**) CCK8 assay was used to detect the effect of BANF1 knockdown on the viability of BGC-823 cell line. (**E**, **F**) The effect of BANF1 knockdown on the proliferative capacity of MKN-45 and BGC-823 cells was detected by plate clone formation assay. (**G**, **H**) Effects of BANF1 knockdown on the migration ability of MKN-45 and BGC-823 cells were detected by wound healing assay. (**I**, **J**) Effect of BANF1 knockdown on the migration and invasion of MKN-45 and BGC-823 cells by Transwell assay. All experiments were repeated at least three times. ^***^p < 0.001.

Silencing BANF1 remarkably impedes the proliferation, migration, and invasion of GC cells. To examine the effect of BANF1 on the phenotypic attributes of these cells, we used lentiviral vectors to establish stable cell lines with downregulated BANF1 expression. This intervention resulted in a substantial reduction in both the mRNA (*p* < 0.001) and protein levels of BANF1 in MKN-45 and BGC-823 cell lines, as shown in Figure 8A, 8C. Furthermore, a cell viability assay decisively underscored the diminished proliferative capacity of MKN-45 and BGC-823 cells after BANF1 knockdown in contrast to their blank control counterparts, as shown in Figure 8B, 8D (*p* < 0.001). The plate clone formation assay also showed that the knockdown of BANF1 inhibited the proliferation of GC cells (Figure 8E, 8F; *p* < 0.001). Elaborating on the migratory attributes, wound healing assays unveiled a significant attenuation in the migration capability of GC cells following BANF1 knockdown, an observation vividly captured in Figure 8G, 8H (*p* < 0.001). Transwell assays further reinforced these findings, affirming that the knockdown of BANF1 resulted in a marked impediment to the migration and invasion capacities of GC cells, as shown in Figure 8I, 8J (*p* < 0.001).

### Knockdown of BANF1 significantly inhibited the growth of subcutaneous tumors in nude mice

**Figure 9:**
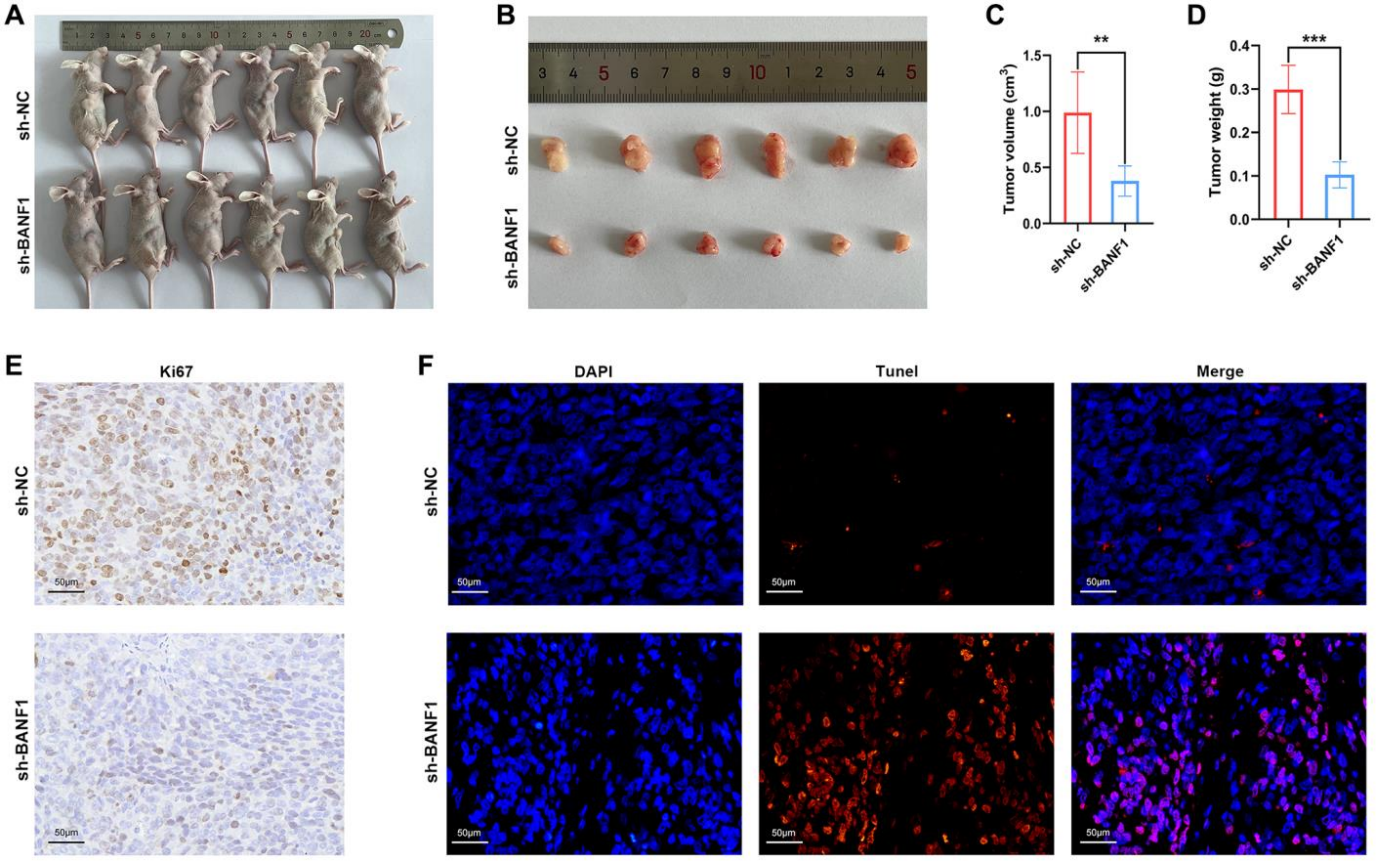
**Knockdown of BANF1 inhibited tumor growth in in vivo experiments.** (**A**) Photographs of nude mice injected with BANF1 knockdown MKN-45 (*n* = 6) and blank control MKN-45 cells (*n* = 6). (**B**) Photographs of subcutaneous tumors of nude mice in the knockdown group (*n* = 6) and control group (*n* = 6). (**C**, **D**) Comparison of subcutaneous tumor volume and weight of nude mice in knockdown and control groups. (**E**) Immunohistochemical staining of Ki67 in subcutaneous tumors of nude mice. (**F**) TUNEL staining of subcutaneous tumors in nude mice. All experiments were repeated at least three times. ^**^p < 0.01, ^***^p < 0.001.

To elucidate the influence of BANF1 expression on tumorigenesis and tumor growth, we performed *in vivo* experiments using a cell line-derived xenograft (CDX) model. In this model, both the control and experimental groups received injections of MKN-45 cells treated with sh-NC and sh-BANF1, respectively, enabling a meticulous assessment of subcutaneous tumor development. The results distinctly indicated a noteworthy suppression of tumor growth after BANF1 knockdown, with the knockdown group showing substantially reduced tumor volume and weight in comparison to the control group, as depicted in Figure 9A–9D (*n* = 6). Furthermore, immunohistochemical staining of mouse tumors provided deeper insights into the influence of BANF1 on tumor proliferative capacity. The results unequivocally revealed a conspicuous reduction in Ki67 expression within the BANF1 knockdown group as opposed to that in the control group, as shown in Figure 9E. Subsequently, the terminal deoxynucleotidyl transferase (TdT)-mediated dUTP nick-end labeling (TUNEL) assay was deployed to examine the impact of BANF1 on apoptosis in GC cells, where the findings showcased a markedly heightened level of tumor apoptosis after BANF1 knockdown relative to the control group, as depicted in Figure 9F.

## DISCUSSION

The incidence and fatality rates of GC remain high, often leading to delayed surgical interventions due to late-stage diagnoses, resulting in unfavorable prognoses [13]. The current therapeutic landscape for GC emphasizes personalized and precision-oriented approaches, including surgery, chemotherapy, radiotherapy, targeted therapy, and immunotherapy, all aimed at improving patient outcomes [14]. GC is a highly heterogeneous disease, leading to varying treatment responses among patients [15]. The molecular subtype-based classification has paved the way for individualized therapeutic strategies, offering a promising avenue for identifying novel therapeutic targets [16].

Various analytical techniques, such as WGCNA, LASSO, SVM-RFE, and RF, have been widely employed to explore disease markers and potential therapeutic targets. In a study by Zhang et al. [17], the WGCNA and RF algorithms were used to identify marker genes in acute pancreatitis. Feng et al. [18] utilized the LASSO algorithm to identify key genes for constructing a diagnostic model for coronary artery disease. A recent study [19] employed LASSO and SVM methods to identify metastatic biomarkers in colorectal cancer. These studies utilized either one or both machine learning algorithms, and the results validated the effectiveness and reliability of these approaches. To identify new ways to treat GC, we jointly applied these four algorithms in an exhaustive exploration of GC transcriptome data derived from the GEO database. Ultimately, we identified three genes (BANF1, ADH7, and TMEM27). The KM plotter database, known for its extensive data and reliable results, has been widely used for studying the correlation between gene expression and the prognosis of various cancers, mainly breast, ovarian, lung and stomach cancers [20–23]. Survival analysis of the three characterized genes revealed a significant correlation between elevated BANF1 expression and poor prognosis (including OS, FP, and PPS) in patients with GC. Our study, based on TCGA database, revealed substantial overexpression of BANF1 in more than a dozen different cancer types, including GC. Zhang et al. [10] showed that BANF1 expression is elevated in breast cancer and is correlated with lymph node metastasis. Jin et al. [11] showed that BANF1 expression is elevated in esophageal cancer. We also verified the high expression of BANF1 in GC tissues and cells, which is consistent with the results of previous bioassay analyses. Mao et al. [12] showed that inhibition of BANF1 in cervical cancer inhibited the proliferation, migration, and invasion of cervical cancer cells, and Ren et al. [24] showed that inhibition of BANF1 inhibited the proliferation and migration of esophageal cancer cells. Recent clinical studies have shown that BANF1 expression is elevated in patients with pancreatic cancer and short survival times [25]. There are very few studies on the mechanism of action of BANF1 in cancer, and most of the studies have only included expression- and cellular-level studies. Our study demonstrated that BANF1 inhibition significantly suppressed the proliferation, migration, and invasion of GC cells. In addition, we performed animal experiments and showed that BANF1 knockdown significantly inhibited the growth of subcutaneous tumors in nude mice, further demonstrating the protumorigenic role of BANF1 in GC. However, the exact molecular mechanism of BANF1 involvement in cancer remains unclear. We analyzed BANF1 expression and immune infiltration in GC and found that BANF1 expression negatively correlated with immune infiltration. Immune infiltration in the TME plays a critical role in tumor development and affects patient prognosis; an effective immune response can inhibit tumor progression [26]. This may also be one of the reasons for the poor prognosis in patients with high BANF1 expression.

Immunotherapy is a highly effective approach to cancer treatment. However, its clinical efficacy varies due to individual differences and the development of drug resistance. Cyclic GMP-AMP synthase (cGAS), a key player in the stimulator of the interferon genes (STING) pathway, has gained widespread attention for its critical role in promoting antitumor immune responses [27–30]. The cGAS-STING pathway enhances the cytotoxicity of immune effectors, including T and natural killer cells, by initiating downstream signaling events, particularly type I interferons. This activation significantly improves the efficacy of immunotherapy [31]. The exposure of large amounts of nuclear DNA to the cytoplasm and its interaction with cGAS effectively trigger an immune response. BANF1, vital for maintaining the structural integrity of the nuclear membrane, competitively binds to cGAS [32]. Consequently, BANF1 exerts a regulatory effect on the cGAS-STING pathway. Recent studies have shown that the upregulation of BANF1 results in reduced expression of cGAS proteins, hindering the innate immune response through the cGAS-STING cascade. Conversely, inhibiting BANF1 upregulates cGAS protein expression, thereby triggering an antiviral immune response [33]. We investigated the effect of BANF1 on immunotherapy efficacy (Supplementary Figure 3) and showed that there was a significant difference in the expression of BANF1 in the response and non-response groups in the iMvigor210 cohort. This suggests that BANF1 may be an important target for modulating the effects of immunotherapy. A recent study exploring the role of BANF1 in tumor immunity revealed that the knockdown of BANF1 increased immune cell infiltration into the tumor microenvironment. This resulted in a significant inhibition of melanoma and colon tumor growth in immunocompetent mice. Moreover, BANF1 knockdown enhanced the therapeutic effect of anti-PD-1 [34]. Despite these findings, the precise role of BANF1 in GC immunotherapy remains unknown. Conventional therapies have demonstrated limited efficacy in treating middle- and advanced-stage GC, prompting the exploration of immunotherapy as a viable alternative [35]. Clinical observations underscore the potential of immune checkpoint inhibitors as stand-alone or combination therapy options, leading to improved prognostic outcomes in selected patient cohorts [36, 37]. Our analysis, based on the TCGA database, indicated a negative correlation between elevated BANF1 expression in GC tumor tissues and immune cell infiltration within these tissues. This suggests that increased BANF1 expression may inhibit anti-tumor immune responses within GC. Combining these findings with existing literature, targeting BANF1 has emerged as a promising avenue to enhance GC responsiveness to immunotherapeutic interventions.

Our investigation has encountered several limitations. First, we have not fully elucidated the precise molecular mechanisms that govern the inhibition of GC progression following BANF1 knockdown. Second, the limited availability of clinical specimens has impeded our ability to conduct a comprehensive analysis of the relationship between BANF1 expression and clinical parameters. Lastly, the effectiveness and safety of therapeutic interventions targeting BANF1 in the clinical milieu await rigorous empirical verification. In summary, despite these limitations, our study presents a promising avenue for the prospective treatment of GC through the strategic targeting of BANF1.

## CONCLUSION

Our study used WGCNA in conjunction with three distinct machine-learning algorithms to identify a triad of signature genes implicated in the pathogenesis of GC. These genes—BANF1, ADH7, and TMEM27—emerged as crucial players in this disease. Notably, BANF1 exhibits heightened expression levels in GC and is closely associated with an unfavorable prognosis in individuals affected by this malignancy. Additionally, our study provides evidence of the impact of BANF1 on the proliferation and migratory capacity of GC cells. This evidence is drawn from both *in vivo* and *in vitro* experimental paradigms. However, it is important to emphasize that the mechanistic intricacies underlying the role of BANF1 in the genesis and progression of GC require further comprehensive elucidation in future studies.

## MATERIALS AND METHODS

### GC transcriptome data and tissue samples

Three high-throughput sequencing datasets about GC (GSE54129, GSE118916, and GSE65801) were acquired from the GEO repository (https://www.ncbi.nlm.nih.gov/geo/), while cancer transcriptome data from the TCGA database were procured using the GDC download tool. The data were meticulously organized using the R software (v4.1.2). Twenty-three paired fresh GC tissues and corresponding paracancerous tissues were surgically excised and collected under the auspices of the Medical Ethics Committee of the First Affiliated Hospital of Anhui Medical University. The samples were preserved in liquid nitrogen for subsequent use.

### Identify differentially expressed genes

Transcriptome data encompassing a cohort of 162 GC samples were meticulously curated through a process involving the integration and harmonization of batch effects within the GSE54129 and GSE118916 datasets using the sva R package. PCA was performed, and the results were visualized using the ggplot2 package. To elucidate the intricate disparities in molecular expression patterns between tumor and normal tissues, we subjected the transcriptome data to rigorous analysis using the Limma package. Differential gene expression profiling was performed, adhering to stringent criteria, with a focus on genes exhibiting a |log2fold change (FC)| >2 and a false discovery rate (FDR) <0.01. Visualization of the DEGs within GC tissues was accomplished by generating heat maps using the pheatmap R package.

### Weighted correlation network analysis

WGCNA is a pivotal biological approach used to explore gene interrelationships across multiple samples [38–40]. This method discerns clusters of genes that exhibit high inter-correlations, thus forming cohesive modules. Moreover, we aimed to identify biomarkers and prospective therapeutic targets by scrutinizing the correlation between these gene modules and the disease phenotype. Gene expression data were meticulously processed using the WGCNA R package, culminating in the creation of a heat map illustrating the interplay between gene modules and clinical information. Among the diverse spectrum of gene modules identified, paramount attention was paid to the one exhibiting the highest correlation with GC, designating it as the pivotal module. Subsequently, hub genes were methodically sieved in accordance with stringent criteria, whereby they were mandated to possess a GS exceeding 0.5, and MM surpassing the threshold of 0.8.

### Identification of hub genes related to GC

Machine learning, with its capacity to adeptly navigate intricate genomic data and discern more precise associations with diseases, offers an efficient avenue for exploring disease-related genes. In this study, we harnessed a trio of machine learning algorithms, namely, LASSO regression analysis, Random Forest (RF), and Support Vector Machine Recursive Feature Elimination (SVM-RFE) [41–43], to conduct an additional layer of scrutiny of the previously identified DEGs. The ensuing step involved the convergence of the key genes identified through WGCNA with a set of differential genes gleaned through the application of the three machine-learning algorithms. This amalgamation yielded a distinctive ensemble of genes that are characteristic of GC.

### Evaluation of characterization genes

To assess the predictive performance of the three identified genes in diagnosing GC, we conducted an ROC analysis of the transcriptomic data. This analysis was performed using the pROC package, with the AUC serving as the benchmark for evaluating diagnostic efficacy. Subsequently, we investigated the prognostic relevance of these three genes in the context of GC using the Kaplan–Meier plotter database (https://kmplot.com/analysis). Patients were stratified into high- and low-expression groups based on median gene expression values and survival curves, encompassing OS, FP, and PPS, which were constructed. Differences in survival outcomes between the two groups were rigorously scrutinized using the log-rank test for statistical comparison.

### Single-cell RNA sequencing data analysis and immunocorrelation analysis

Retrieve the scRNA-seq datasets pertinent to GC from the TISCH (database http://tisch.comp-genomics.org/). Employing the “FindClusters” function with a resolution parameter set at 0.2, we ascertained cellular clusters within the datasets. Subsequently, we visualized the data by employing t-SNE, we visualized the data. To identify the DEGs within each of these clusters, we leveraged the FindAllMarkers algorithm and selected the top 10 genes with the most significant differences as markers for each cell cluster. Cell annotation was performed manually using these markers. For data dimensionality reduction analysis, we opted for the UMap method and visualized the expression of BANF1 across various cell types. Furthermore, in the TCGA GC samples, we computed immune infiltration using 24 immune cell markers and the single sample gene set enrichment analysis (ssGSEA) algorithm in the GSVA R package. The Spearman method was employed to analyze the correlation between BANF1 expression and the degree of immune infiltration, and the results were depicted as a Laplace plot using the ggplot2 R package. We categorized the samples into high and low BANF1 expression groups based on the median BANF1 expression value, and compared immune infiltration between these groups using the Wilcoxon rank-sum test. To gauge the impact of BANF1 expression on the immune microenvironment, we calculated the immune, stromal, and ESTIMATE scores for GC samples using the ESTIMATE R package. Higher scores indicated a greater abundance of these components within the tumor microenvironment. Differences in scores between the groups characterized by high and low BANF1 expression were assessed using the Wilcoxon rank-sum test. The renal cell carcinoma and melanoma immunotherapy cohorts were obtained from the GEO website, the urothelial carcinoma dataset was downloaded from the IMvigor210 (http://research-pub.gene.com/IMvigor210CoreBiologies), and the gastric cancer immunotherapy cohort (PRJEB25780) was obtained from Tumor Immune Dysfunction and Exclusion (TIDE). The patients were divided into response group and non-response group, and the difference of BANF1 expression between the two groups was compared.

### Gene set enrichment analysis

To identify the enriched pathways associated with BANF1 expression, GSEA was performed on the BANF1 high and low expression groups using GSEA software (v4.2.1) and gene set (C2. Cp.kegg. V7.4. Symbols GMT). The top five pathways were selected based on the *p* < 0.05 criterion.

### Pan-cancer expression analysis of BANF1

Data retrieval and curation involved the acquisition of information from TCGA database (https://portal.gdc.cancer.gov/), encompassing transcript data expressed in transcripts per kilobase million (TPM) format for 33 tumor types. Groups consisting of fewer than three samples or those displaying zero variance were excluded from subsequent analyses. To discern disparities in BANF1 expression, the Wilcoxon rank-sum test was used to compare the expression between the cancer and paracancer groups.

### RNA extraction and real-time quantitative polymerase reaction

In the investigation of GC tissues and adjacent non-tumor tissues, total RNA extraction was performed utilizing the HiPure Universal RNA Kit (Magen, Shanghai, China), following the manufacturer’s guidelines. Subsequently, the concentration and purity of the RNA samples were assessed employing a Nanodrop 2000 (Thermo Fisher Scientific, USA). The transcribed cDNA was generated from the extracted RNA using the PrimeScript RT kit (Vazyme, Nanjing, China). The quantification of BANF1 mRNA expression was conducted through the 2^−ΔΔCT^ method, utilizing GAPDH as an internal control.

### Western blotting

Tissues and cells underwent lysis using a phenylmethanesulfonyl fluoride (PMSF)-containing lysis buffer (Beyotime, Shanghai, China) to extract proteins. After electrophoresis and membrane transfer, the proteins were sealed with 5% skim milk for 1.5 hours, and the membrane was incubated with anti-BANF1 (1:1000, Abcam, UK) or anti-GAPDH (1:50000, Proteintech, China) at 4°C overnight. After washing with Tris-buffered saline containing 0.1% Tween 20 (TBST), the membranes were immersed in anti-rabbit/mouse secondary antibodies (1:10000, Proteintech, China) for 2 hours at room temperature. Finally, membrane visualization and imaging were achieved using an ECL chemiluminescence system (Tanon, Shanghai, China).

### Immunohistochemistry

Immunohistochemical staining was used to detect ki67 expression in subcutaneous tumors from nude mice. Samples were embedded in paraffin to prepare paraffin sections, which were dewaxed, hydrated, and antigenically repaired according to standard procedures. Sections were titrated with anti-ki67 antibody (1:1000, CST, USA) and incubated at 4°C overnight. After washing, enzyme-labeled sheep anti-rabbit IgG polymer was added dropwise and incubated at 37°C for 20 min. Incubate with freshly prepared diaminobenzidine chromogenic solution and finally with hematoxylin staining solution.

### Immunofluorescence

The assessment of BANF1 protein expression and subcellular localization was conducted in MKN-45 and BGC-823 cells. Following fixation with 4% paraformaldehyde, cells were subjected to overnight incubation with the BANF1 primary antibody at 4°C. Subsequent to thorough washing, Fluorescent secondary antibody was applied and incubated at room temperature for 1 hour. The cellular nuclei were counterstained with 4′,6-diamidino-2-phenylindole (DAPI) for a duration of 10 minutes.

### Cell culture and stabilized cell line construction

Human gastric cancer cell lines (AGS, HGC-27, MKN-45, MGC-803, and BGC-823) and the normal gastric mucosal epithelial cell line GES-1 were sourced from the American Type Culture Collection (ATCC). The cells were cultured in 1640 medium (Gibco), supplemented with fetal bovine serum and penicillin-streptomycin. Incubation occurred at 37°C with a CO_2_ concentration maintained at 5%. For transfection, GC cells and co-transfection reagents (GeneChem, Shanghai, China) were added to 12-well plates at specific ratios. Notably, MKN-45 and BGC-823 cells were utilized for gene knockdown experiments. Stably expressing cells were subsequently selected through screening with 5 μg/ml puromycin.

### Cell proliferation capacity assay

The cell proliferative capacity was determined using a cell counting kit-8 (CCK8) assay and a plate clone formation assay. Stably transformed cells were added to 96-well plates at 3000 cells/well. The absorbance at 450 nm was measured using an enzyme marker after adding the CCK8 reagent at 0 h, 24 h, 48 h, 72 h, and 96 h respectively. The plate clone formation assay was performed by adding 1000 cells/well to 6-well plates and changing the medium every 3 d for a total of approximately 10 d. Cells were fixed with paraformaldehyde and stained with crystal violet after clone formation.

### Wound healing assays

GC cells were cultured in 6-well plates, and when the cells were fully grown, the tip of a pipette was used to make a scratch approximately 2 mm wide. Low-serum medium containing 2% fetal bovine serum was added, and the culture was continued. Scratch healing was observed at 0 h and 24 h.

### Transwell assay

Migration and invasion capabilities of gastric cancer (GC) cells were assessed using the Transwell assay. Cells were suspended in a serum-free medium, with 30,000 cells added to the upper layer of each chamber. The lower layer of the chamber received a complete medium containing 10% FBS. For the assessment of cell invasiveness using the Transwell assay, Matrigel (BD, USA) was added to the chambers beforehand, while the Transwell assay without Matrigel was employed to evaluate the migration ability of the cells. Following a 48-hour incubation period, the chambers were carefully removed, washed with PBS, fixed using 4% paraformaldehyde, and subsequently stained with a crystal violet staining solution.

### Cell line-derived xenograft

The immunodeficient mice used in this study were purchased from Empharmatech (Jiangsu, China). Breeding conditions and all operational procedures were performed in accordance with the requirements of the Animal Ethics Committee of Anhui Medical University. We selected 4-week-old nude mice for experiments after acclimatization in a specific pathogen-free (SPF) environment for seven days. Stable BANF1 knockdown MKN-45 cells and MKN-45 control cells were injected subcutaneously into nude mice, and the number and volume of cells injected into each nude mouse were 5 million and 200 µl. The volume of subcutaneous tumors in the nude mice was measured every two days, and the formula for calculating the volume of the tumors was V = ab2/2. The mice were sacrificed at the end of the experiment and disposed of.

### TUNEL assay for apoptosis detection

The principle of apoptosis detection by TUNEL assay is that when DNA breaks during apoptosis, the exposed 3′-OH is labeled with TMR-5-dUTP catalyzed by TdT, which can be fluorescently microscopically observation. Paraffin sections of nude mouse subcutaneous tumors were stained using a one-step TUNEL apoptosis detection kit (Servicebio, Wuhan, China).

### Statistical analysis

Disparities in molecular expression between tumor and normal tissues in publicly available data were assessed through the Wilcoxon rank-sum test. Kaplan–Meier survival curves were employed to illustrate the prognostic differences between high and low BANF1 expression groups, with log-rank tests utilized for inter-group comparisons. The correlation between BANF1 expression and immune cell infiltration was scrutinized using Spearman’s method, and variations in immune infiltration results between high and low BANF1 expression groups were evaluated through the Wilcoxon rank-sum test. Differences in BANF1 expression across various cancer types were examined using the Wilcoxon rank-sum test. All bioinformatics analyses were executed using R software (v4.1.2). Experimental procedures were replicated a minimum of three times. Western blot (WB) results were quantified with ImageJ software, and inter-group differences were analyzed using the *t*-test in GraphPad Prism 8.02. A significance threshold of *p* < 0.05 was applied. Bar graphs depict the mean ± standard deviation (SD) based on the outcomes of the three experiments.

## Supplementary Materials

Supplementary Figures
