# Supplementary material for: Identification of BANF1 as a novel prognostic biomarker in gastric cancer and validation via in-vitro and in-vivo experiments
Source: Aging (Albany NY). 2024 Jan 22;16(2):1808–28. doi: 10.18632/aging.205461 (PMC10866416; doi:10.18632/aging.205461)
Supplement: Supplementary Figures [file aging-16-205461-s001.pdf]

SUPPLEMENTARY FIGURES

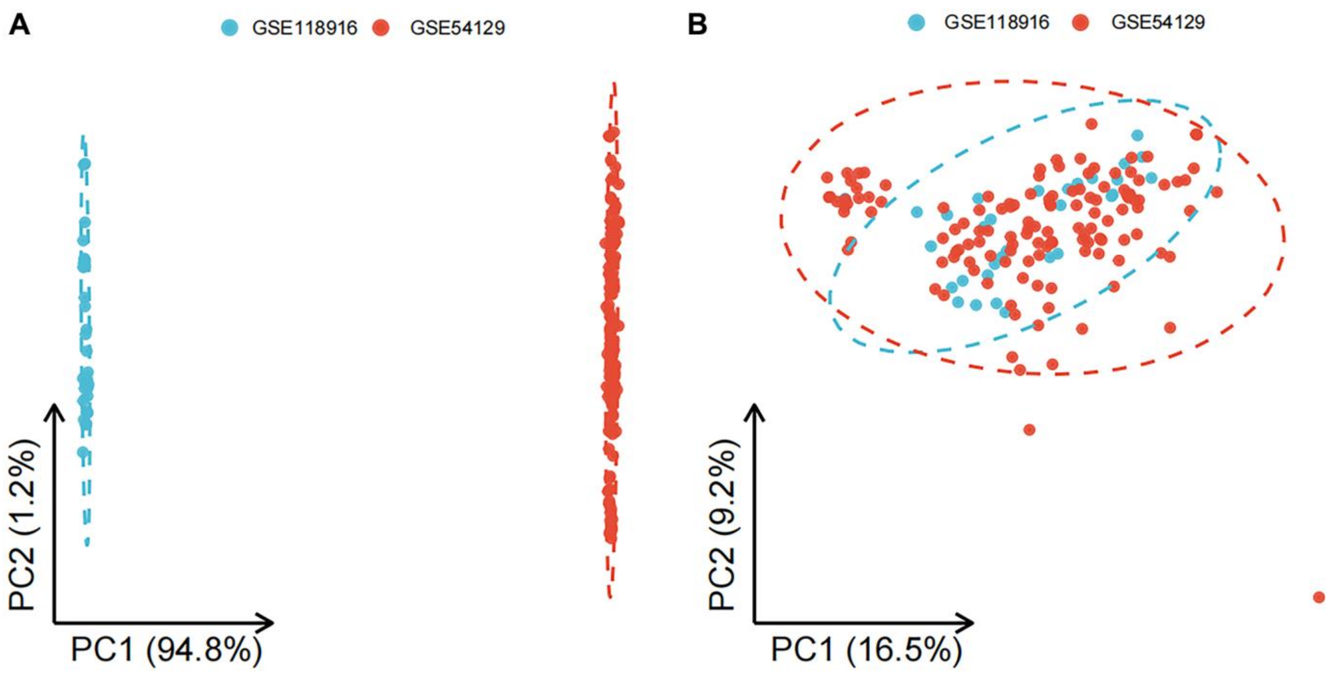

Supplementary Figure 1. Principal component analysis to view the differences between samples. (**A**) Difference between raw data of GSE118916 and GSE54129. (**B**) Expression of data of GSE118916 and GSE54129 after removing batch effects.

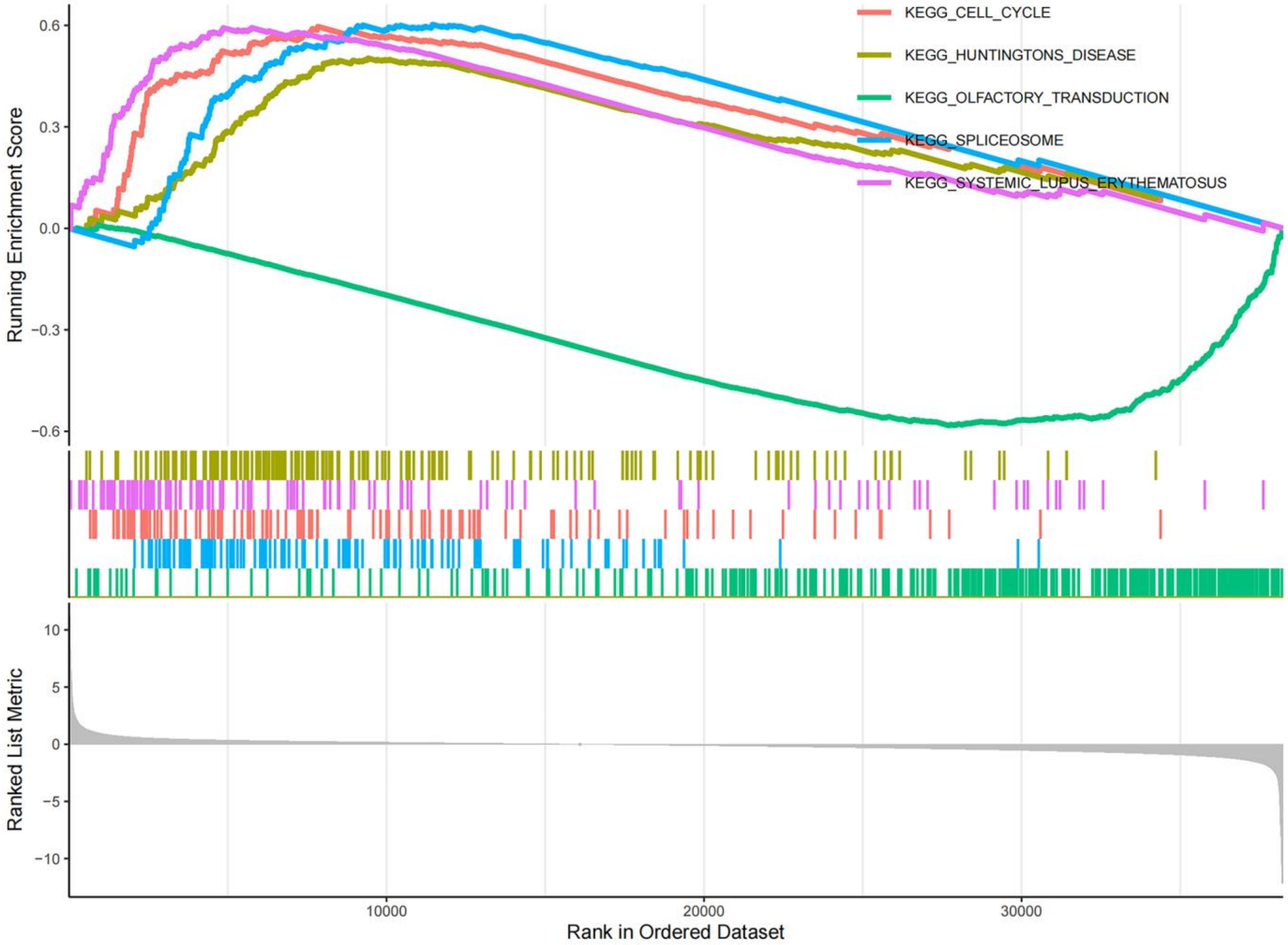

Supplementary Figure 2. Gene set enrichment analysis. The samples were divided into two groups according to the high and low expression of BANF1, the differential genes between the two groups were calculated, and the signaling pathways in which BANF1 might be involved were speculated based on the analysis of the up- and down-regulation of the differential genes.

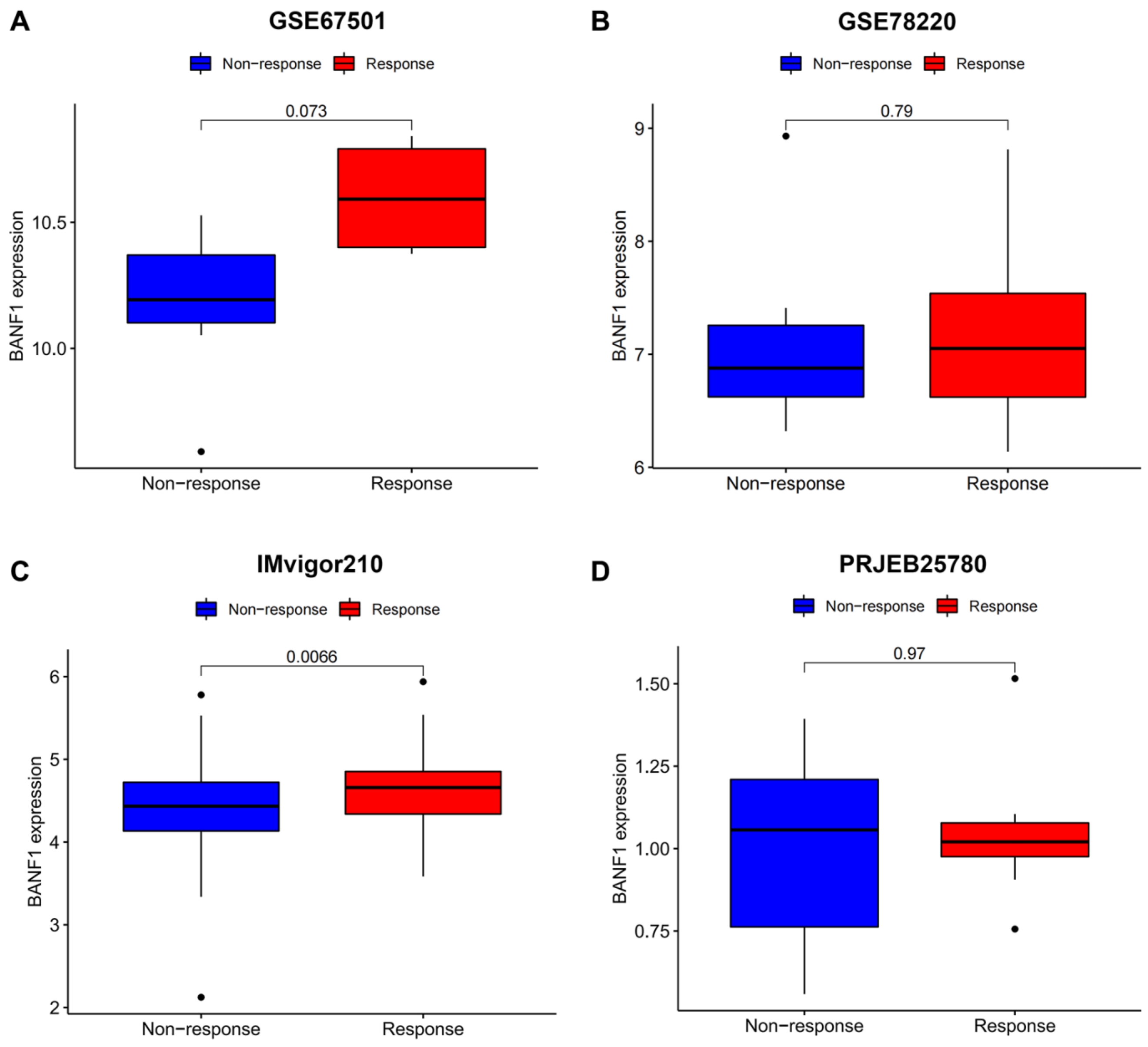

Supplementary Figure 3. Effect of BANF1 expression level on immunotherapy. Differences in BANF1 expression in renal cell carcinoma (**A**), melanoma (**B**), urothelial carcinoma (**C**), and gastric cancer (**D**) immunotherapy cohorts between response and non-response groups.
